# Breaking barriers in peritoneal fibrosis: extracellular vesicles as promising diagnostic and therapeutic strategy

**DOI:** 10.1080/07853890.2026.2659509

**Published:** 2026-04-22

**Authors:** Lichao Zhong, Bohua Zhang, Yupei Li, Baihai Su, Ruoxi Liao

**Affiliations:** Department of Nephrology, Institute of Kidney Diseases, West China Hospital of Sichuan University, Chengdu, China

**Keywords:** Peritoneal fibrosis, extracellular vesicles, exosomes, peritoneal dialysis, therapeutic target

## Abstract

**Background:**

Peritoneal dialysis is an essential therapy for end-stage kidney disease, but its long-term efficacy is severely limited by peritoneal fibrosis. Driven by mesothelial-mesenchymal transition, inflammation and angiogenesis, this pathological process leads to treatment failure and increased mortality. The urgent need for effective therapies has shifted research focus to extracellular vesicles—phospholipid bilayer nanovesicles that shuttle bioactive cargoes to modulate pathogenic pathways, positioning them as key mediators in peritoneal fibrosis.

**Discussion:**

This review synthesises preclinical evidence to evaluate the dual diagnostic and therapeutic potential of extracellular vesicles. By analysing their cellular sources and regulatory roles in fibrotic pathways, we highlight their promise as both non-invasive biomarkers and targeted therapeutic agents for peritoneal fibrosis. It further integrates these mechanistic insights with critical translational hurdles, including unstandardised technical preparation, unclear *in vivo* biodynamics and unconfirmed long-term safety in clinical translation.

**Conclusion:**

Extracellular vesicles hold great potential as novel non-invasive biomarkers and targeted therapeutic agents for peritoneal fibrosis. This review outlines a developmental roadmap for extracellular vesicles-based strategies by combining mechanistic findings with translational challenges. Addressing these critical gaps will be pivotal to unlocking the clinical value of extracellular vesicles, preserving peritoneal membrane function and improving long-term outcomes for relevant patients.

## 1. Introduction

Chronic kidney disease (CKD) has become a serious public health challenge in the twenty-first century, with an increased disease burden closely linked to the global prevalence of key metabolic risk factors, such as hypertension, diabetes, and obesity [1–3]. As the disease progresses to end-stage kidney disease (ESKD), a terminal stage with a rapid decline in the glomerular filtration rate (GFR), renal replacement therapy (RRT) is critical for patient survival [4–6]. The primary RRT modalities are haemodialysis (HD), peritoneal dialysis (PD), and kidney transplantation (KT). Although KT offers the best clinical outcomes, donor scarcity leaves dialysis as the mainstay for most ESKD patients [7–9]. As a key RRT modality, PD relies on the intrinsic transport properties of the peritoneal tissue to remove excess water and uraemic toxins [10,11]. Compared with HD, PD offers several advantages, including procedural simplicity, home-based therapy, cost-effectiveness, and improved survival [11,12].

Despite its therapeutic potential, PD remains underutilised globally, currently serving merely 11% of the dialysis population [12,13]. A major obstacle to its wider adoption is peritoneal fibrosis (PF), characterised by progressive deterioration of peritoneal structure and function [10,14]. Within 24 months of PD initiation, signs of PF are observed in 50–80% of patients [15]. Pathologic factors, including nonphysiological dialysate components (e.g. acidic pH, hypertonic glucose, and glucose degradation products), recurrent peritonitis, and oxidative stress, synergistically alter peritoneal integrity [16–19]. This disruption, mediated through peritoneal mesothelial–mesenchymal transition (MMT), submesothelial extracellular matrix (ECM) deposition, chronic inflammation, and pathological angiogenesis, drives the progression of PF [16–20]. This fibroproliferative process progressively alters solute transport efficiency, gradually dissipates the osmotic gradient and accelerates the loss of residual renal function, ultimately leading to ultrafiltration failure and inevitable discontinuation of dialysis [21–23]. In addition, the progression of severe peritoneal fibrosis to encapsulating peritoneal sclerosis (EPS) is a rare but severe complication in PD patients, with a reported prevalence ranging from 0.4% to 8.9% across North America, Europe, Asia, and Oceania [15,24–26]. The increasing prevalence of PF in long-term PD necessitates innovative therapies targeting its multifactorial pathogenesis.

**Figure 1.**
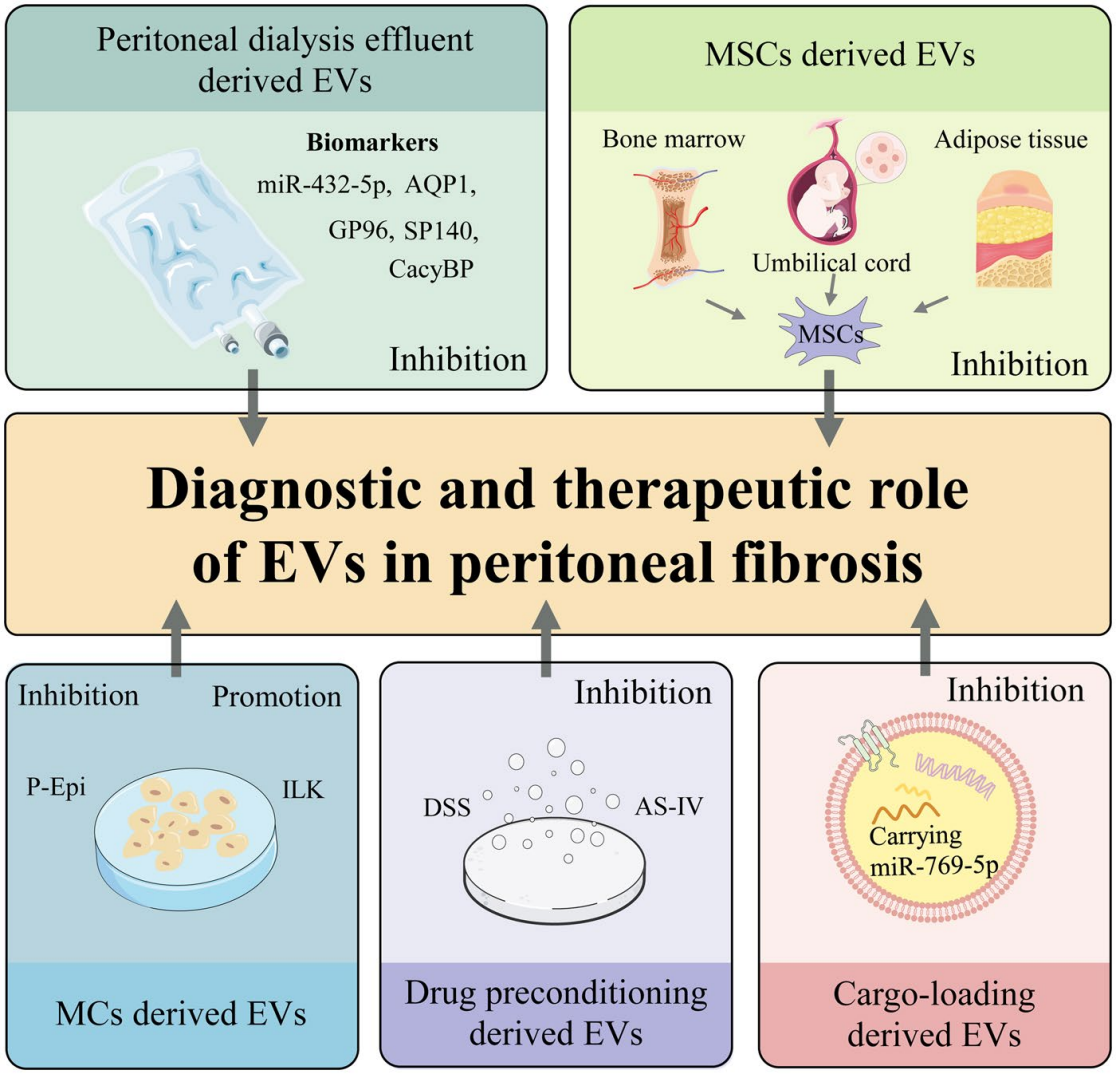
Schematic overview of EV sources and their roles in peritoneal fibrosis. Extracellular vesicles (EVs) derived from various sources—including mesothelial cells (MCs), peritoneal dialysis effluent, mesenchymal stem cells (MSCs), drug-preconditioned cells, and cargo-loading EVs—contribute to both the diagnosis and treatment of peritoneal fibrosis. Arrows indicate the functional contribution of each EV source. MC-derived EVs exhibit dual roles (pro-fibrotic *via* ILK, anti-fibrotic *via* P-Epi), whereas EVs from other sources primarily exert inhibitory effects. Peritoneal dialysis effluent-derived EVs also serve as potential diagnostic biomarkers (e.g. miR-432-5p, AQP1, GP96, SP140, CacyBP). Abbreviations: EVs: extracellular vesicles; MSCs: mesenchymal stem cells; MCs: mesothelial cells; P-Epi: human epithelial-like peritoneal mesothelial cells; ILK: integrin-linked kinase; DSS: Danshenshin; AS-IV: astragaloside IV; miR-432-5p: microRNA-432-5p; AQP1: aquaporin 1; GP96: glycoprotein 96; SP140: speckled protein 140; CacyBP: calcyclin-binding protein; miR-769-5p: microRNA-769-5p.

Extracellular vesicles (EVs)—nanoscale lipid bilayer particles (30–5,000 nm) encompassing exosomes, microvesicles (MVs), and apoptotic bodies—offer new insights into molecular mechanisms of PF. Derived from diverse cell types and widely distributed in bodily fluids, EVs transport bioactive molecules (proteins, nucleic acids, lipids) to facilitate intercellular communication, positioning them as promising therapeutic agents for coordinating tissue repair, immune regulation, and homeostasis [27–30]. Given these properties, EVs have garnered increasing attention as potential regulators of PF pathogenesis. This review synthesises current knowledge on the contributions of EVs to PF progression in patients undergoing long-term PD. We focus on exosomes, which are the most extensively studied EV subtype in this context, given their distinctive cargo that underpins both biomarker utility and therapeutic potential. Herein, the general term ‘EV’ is used when original studies do not specify vesicle subtypes. Key topics covered include the pathologic mechanisms of PF, the biological characteristics of EVs, and the diagnostic value, therapeutic roles and underlying mechanisms of EVs in PF. We further discuss challenges associated with harnessing exosomes as drug delivery vehicles and explore future directions for EV-based interventions in PD. A schematic overview of EV sources and their roles in the diagnosis and treatment of PF is presented in Figure 1.

## 2. Pathologic mechanisms of PF

### 2.1. Inflammation and immune cell infiltration

In PD-related PF, bioincompatible dialysate components, infections, and uraemic toxins synergistically provoke an inflammatory response [31,32]. The injured peritoneum recruits immune cells (e.g. macrophages, neutrophils, monocytes, and Th17 cells) to produce cytokines such as interleukin-1β (IL-1β), IL-6, and tumour necrosis factor-α (TNF-α) [33,34]. These inflammatory mediators subsequently trigger macrophage polarisation from a pro-inflammatory M1-dominated phenotype to a profibrotic M2-dominated phenotype [32,35]. In turn, this inflammatory milieu promotes fibroblast proliferation, MMT, and ECM accumulation [34], perpetuating peritoneal damage. Mechanistically, inflammation serves as the initiating trigger: it directly damages mesothelial cells and establishes a microenvironment permissive for subsequent TGF-β-driven fibrogenesis [36].

### 2.2. Transforming growth factor beta (TGF-β) and MMT

MMT is a central effector in PF, primarily driven by TGF‑β1 through dual signalling pathways: (i) The Smad-dependent pathway, mediated by TGF-β receptor type 2 (TβRII), leads to nuclear translocation of phosphorylated Smad2/3/4 complexes [34,37]; (ii) the Smad-independent pathway involves multiple signalling cascades, including extracellular signal-regulated kinase (ERK), p38 mitogen-activated protein kinase (p38 MAPK), phosphoinositide 3-kinase/protein kinase-B (PI3K/AKT), and c-Jun N-terminal kinases (JNK) [10,31,38]. Notably, ERK is a major MMT inducer, whereas p38 MAPK exerts a dual role by promoting MMT and maintaining E-cadherin expression [39,40]. These converging pathways coordinately upregulate the transcription of profibrotic genes (e.g. α-SMA, collagen I/III and fibronectin), and downregulate E-cadherin *via* Snail. Consequently, these changes lead to excessive ECM deposition and subsequent disruption of the peritoneal mesothelial integrity [21,37,38,41]. Among the three core mechanisms, TGF-β-mediated MMT occupies a pivotal position: it is both a key downstream effector of inflammation and an autonomous driver of fibrosis that can amplify its own signalling through positive feedback loops [42].

### 2.3. Angiogenesis and the hypoxic microenvironment

**Figure 2.**
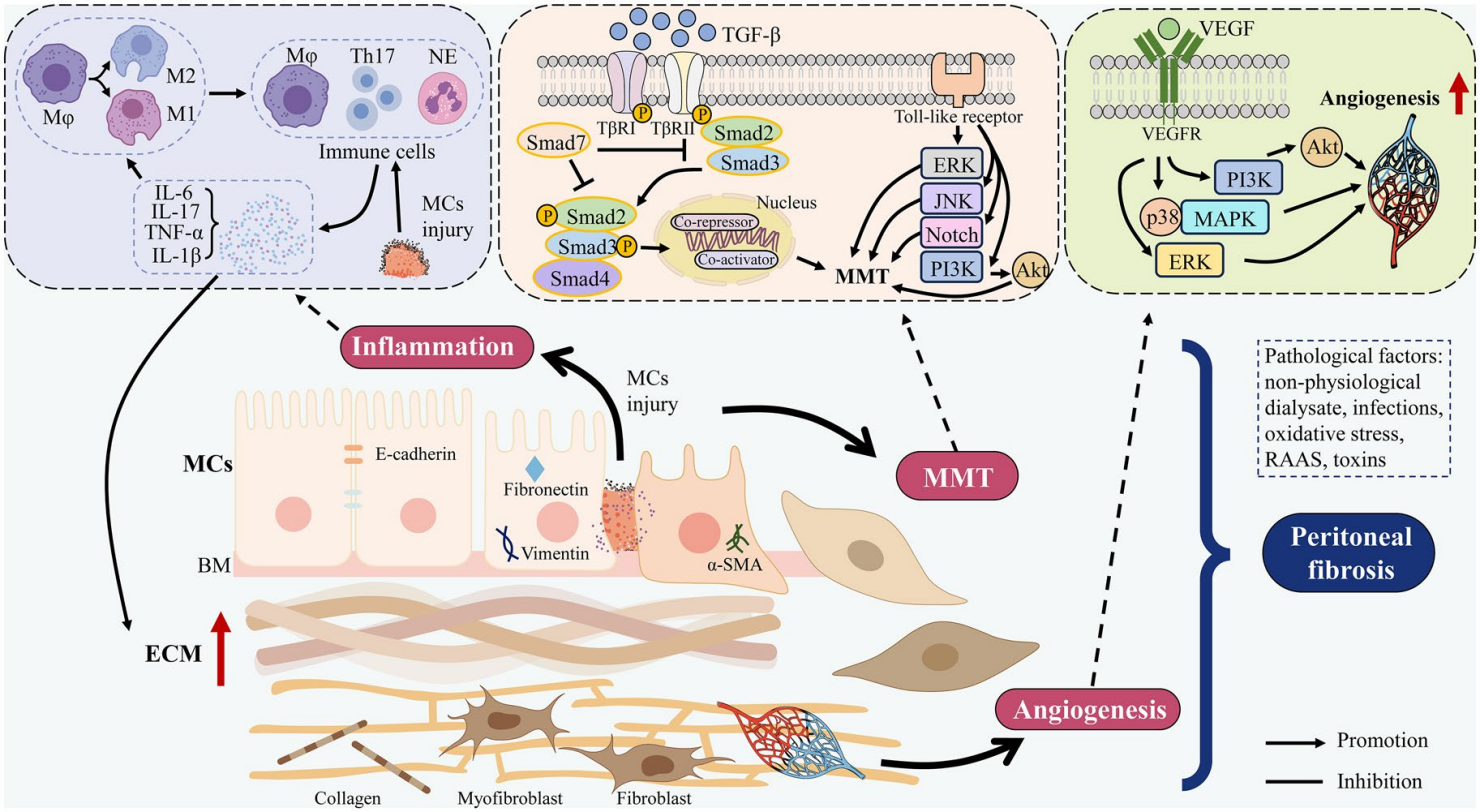
Presents a schematic illustration of the current understanding of the pathologic mechanisms of peritoneal fibrosis. The central module outlines three primary mechanisms—inflammation, mesenchymal–mesenchymal transition (MMT), and angiogenesis—while the upper three panels detail their specific processes and associated signalling pathways. Note: Mφ: Macrophages; NE: neutrophil; ERK: extracellular signal-regulated kinase; JNK: c-Jun N-terminal kinase; PI3K: phosphoinositide 3-kinase; Akt: protein kinase-B; MAPK: mitogen-activated protein kinase; VEGF: vascular endothelial growth factor; VEGFR: vascular endothelial growth factor receptor; MCs: mesothelial cells; BM: basement membrane; ECM: extracellular matrix; MMT: mesothelial–mesenchymal transition.

Pathological angiogenesis drives the peritoneal functional deterioration [43]. A key mediator of this process is the vascular endothelial growth factor (VEGF)/vascular endothelial growth factor receptor (VEGFR) axis, which promotes the formation of immature, fragile, and hyperpermeable vessels [44,45]. Neovascularization is also associated with a pseudohypoxic state in glucose-overloaded peritoneal mesothelial cells. An elevated NADH/NAD^+^ ratio promotes the expression of hypoxia-inducible factor 1 subunit α (HIF-1α), matrix metalloproteinase 2 (MMP-2), and VEGF [46]. Although angiogenesis is not an initiator of fibrosis, it acts as a critical chronic amplifier of disease progression [42]. Figure 2 illustrates the integrated pathologic mechanisms of PF.

Targeting these pathological mechanisms has shown therapeutic promise. For instance, bone morphogenetic protein-7 (BMP-7) counteracts TGF-β1-induced MMT and fibrosis *via* Smad1/5/8 activation and Smad2/3 inhibition [47,48]. Epigenetic regulation has emerged as a central hub in this pathological network. Inhibition of epigenetic modifier enhancer of zeste homolog 2 (EZH2) suppresses pathological angiogenesis *via* the VEGFR2/ERK1/2/HIF-1α axis [49]. Similarly, AZ505, a selective inhibitor of the methyltransferase SET and MYND domain protein 2 (SMYD2), simultaneously mitigates MMT, inflammation, and angiogenesis [50]. Beyond protein-level regulators, non-coding RNAs have also garnered attention. LncRNA AK142426 regulates M2 macrophage polarisation, and its knockdown ameliorated fibrosis and inflammation [51]. Additionally, EVs have emerged as a promising research focus due to their biocompatibility and multifunctional cargo delivery capabilities, warranting further investigation in PF.

## 3. Physiological characteristics of EVs

### 3.1. How are EVs synthesized?

EVs are a heterogeneous population of nanosized lipid bilayer particles released by virtually all cell types. They are classified into several major subtypes based on their biogenesis and size, including exosomes (50–150 nm), microvesicles/ectosomes (100–1,000 nm) and apoptotic bodies (100–5,000 nm) [52,53]. These subtypes have distinct biogenesis pathways: exosomes originate from the endosomal system [54,55], microvesicles bud directly from the plasma membrane [56], and apoptotic bodies are released during programmed cell death [57]. Despite these differences in origin, all EV subtypes transfer bioactive molecules (proteins, lipids, and nucleic acids) to mediate intercellular communication in physiological and pathological processes. Given the well-documented role of exosomes in PF and their high stability in body fluids—key advantages for potential therapeutic and diagnostic applications—this review focuses specifically on exosomes.

### 3.2. What components do EVs contain?

During their biogenesis and release, EVs are loaded with a diverse array of molecular cargoes, including proteins, nucleic acids, lipids, and metabolites [58,59]. As key subtypes of EVs, exosomes, microvesicles, and apoptotic bodies exhibit distinct cargo compositions: exosomes are enriched in selectively sorted microRNAs (miRNAs), long noncoding RNAs (lncRNAs), messenger RNAs (mRNAs), tetraspanins (CD9/CD63/CD81), ESCRT proteins, and heat shock proteins [60–62]; microvesicles carry diverse molecules, including tissue factors, protease-activated receptors, bioactive lipids, and integrins [63]; whereas apoptotic bodies mainly contain chromatin remnants, organelle debris, and DNA fragments [64,65].

### 3.3. What role do EVs play?

As cell-derived nanovesicles, EVs mediate intercellular and interorgan communication through dual mechanisms: they either enter the systemic circulation to regulate distant tissues or remain localised near their secretion sites to facilitate autocrine/paracrine signalling [66–69]. In addition, they act as immune system regulators through antigen-presenting mechanisms and modulation of immune cells such as neutrophils, dendritic cells, and macrophages [70–72]. Pathologically, tumour-derived EVs significantly promote tumour neovascularization by carrying proangiogenic factors such as VEGF, IL-8 and fibroblast growth factor-2 (FGF-2) [73,74]. Therapeutically, EVs accelerate wound healing and tissue regeneration by regulating the macrophage polarisation phenotype and the fibroblast activation process [75]. Furthermore, EVs have dual utility as non-invasive diagnostic tools and reservoirs of disease-specific biomarkers, owing to their unique ability to cross biological barriers and their cargo profiles that reflect cellular pathophysiology [76–78].

### 3.4. What advances have been made in the clinical application of EVs?

The inherent structural versatility and multifunctional properties of EVs establish them as promising therapeutic candidates, with preclinical and clinical trials validating their efficacy across multiple diseases, such as cancer [79,80], cardiovascular diseases [81,82], pulmonary diseases [83,84], and fibrotic diseases [85–87]. Notably, natural EVs exhibit intrinsic biocompatibility, low immunogenicity, and biostability, positioning them for precise delivery of therapeutic drugs [88–90]. Beyond their native applications, EVs can be engineered to acquire enhanced functionalities. Key functionalization techniques can be broadly classified into two categories: (i) indirect modulation of parent cells (e.g. *via* hypoxia, cytokine, drug preconditioning, and gene editing), which endows the secreted EVs with specific molecular cargoes (e.g. miRNAs, proteins or CRISPR-Cas9 components), thereby conferring predefined biological functions [91–94]; (ii) direct engineering of isolated EVs through physical, chemical, or bioengineering approaches to impart tailored functionalities [95,96], such as surface-conjugated targeting ligands for improved tropism [96,97], hybrid membranes for augmented stability [98], and encapsulation of therapeutic nucleic acids, proteins, or gene editing tools for efficient delivery [95]. Through these strategies, EVs gain superior targeting specificity, achieve precise payload deposition at pathological sites, and expand their therapeutic repertoire [99]. Collectively, these advancements have paved the way for exploring EVs as potential biomarkers and therapeutic strategies in PF.

## 4. Potential and significance of EVs as biomarkers in PF

Peritoneal fibrosis begins with mesothelial cell injury and mild inflammation [100], progresses through chronic inflammation, aberrant neovascularization, and culminates in irreversible fibrosis, elevated peritoneal solute transport rate (PSTR), and ultrafiltration failure [41]. Although peritoneal biopsy is the gold standard for assessing the severity of PF [101], its invasiveness restricts its broad utilisation [102]. Consequently, the development of non-invasive diagnostic tools for PF prediction is highly imperative. EVs derived from peritoneal dialysis effluent (PDE-EVs) offer a promising solution because of their repeatable sampling, high stability and disease-specific cargo enrichment, paving the way for non-invasive or minimally invasive diagnostic strategies [78].

A cohort study involving 30 PD patients demonstrated that exosomes containing the water channel aquaporin 1 (AQP1) in peritoneal dialysis effluent serve as biomarkers for peritoneal membrane integrity and dialysis efficiency. These findings suggest that decreased AQP1 release from exosomes in peritoneal dialysis effluent is associated with impaired ultrafiltration, free water transport, and Na-sieving, reflecting mesothelial cell injury and ultrafiltration dysfunction [103]. Carreras-Planella, L. et al. demonstrated that proteomic changes in extracellular vesicles from peritoneal dialysis effluent (PDE-EVs) precede alterations in the peritoneal equilibrium test (PET) [104]. Subsequently, Fang, J. et al. linked PDE-EV proteomics to PSTR and reported that PDE-EV-glycoprotein 96 (GP96) was specifically elevated in patients with a high PSTR, correlating positively with both PSTR and intraperitoneal IL-6 level [105]. Analysis of exosomal miRNAs in peritoneal dialysis effluent from patients with different peritoneal transport statuses revealed that exosomal miR-432-5p expression was significantly elevated in the high peritoneal solute transport rate (PSTR > 0.65) group. Moreover, its expression levels correlated negatively with 4-hour ultrafiltration volume and 4-hour dialysate sodium removal (DSR) [106]. Interestingly, a clinical study revealed that suppressor of cytokine signalling 1 (SOCS1), speckled protein 140 (SP140), and calcyclin binding protein (CacyBP) from peritoneal dialysis effluent may exert a protective effect on peritoneal function, whereas reduced expression of these three proteins leads to impaired peritoneal function and PF. These three indicators can change significantly in the early stages of fibrosis, suggesting potential for early screening. In addition, the sensitivity and specificity of these three indicators for the combined diagnosis of PF exceeded 90% [107].

These findings establish PDE-EVs as promising biomarkers for monitoring changes in peritoneal function, including inflammation, transport status and fibrotic progression. Moreover, the quantitative dynamics of PDE-EVs following therapeutic intervention could serve as pharmacodynamic indicators for precision management. Meanwhile, with the development of single-cell sequencing, electrochemical sensors and other technologies, the detection and screening of EV biomarkers will be more efficient and precise [108,109]. Future studies should further validate the sensitivity, specificity, and clinical utility of EV biomarkers in PD patients to advance non-invasive diagnostics. However, comprehensive profiling of the full spectrum of EV cargo as diagnostic indicators in peritoneal fluid remains limited, particularly for peritoneal fibrosis. The effects of PDE-EVs as biomarkers of PF are summarised below and shown in Table 1.

**Table 1.** PDE-EVs biomarkers for PF.

Cargoes	Species	Cohort size (*n*)	Change	Pathological significance	References
AQP1	Human	*n* = 30	↑	Positively correlated with PD efficiency and predicting ultrafiltration reduction	[103]
PDE-EV-GP96	Human	*n* = 60	↑	Positively correlated with PSTR and IL-6 levels	[105]
miR-432-5p	Human	*n* = 50	↑	Negatively correlated with 4-hour ultrafiltration and 4-hour DSR	[106]
SOCS1	Human	*n* = 90	↓	Early biomarker for PF	[107]
SP140	Human	*n* = 90	↓	Early biomarker for PF	[107]
CacyBP	Human	*n* = 90	↓	Early biomarker for PF	[107]

AQP1: The water channel Aquaporin 1; PD: peritoneal dialysis; GP96: glycoprotein 96; PSTR: peritoneal solute transport rate; DSR: dialysate sodium removal; SOCS1: suppressor of cytokine signalling 1; PF: peritoneal fibrosis; SP140: speckled protein 140; CacyBP: calcyclin binding protein.

## 5. Role of natural EVs from different origins in PF

Natural EVs from various sources (such as mesenchymal stem cells, macrophages and mesothelial cells) participate in PF pathogenesis, as summarised in animal experiments and cellular studies presented in Tables 2 and 3.

**Table 2.** Animal experiments on the role of extracellular vesicles in peritoneal fibrosis.

Origin of EVs	Cargo	Administration route	Species; sex; age; weight	Sample sizes (*n*)	Establishment of PF models	Dosage; timing	Results	Mechanisms; pathways	References
BMSC	/	IP	C57BL/6 J mice; male; 3 months; 28–30 g	*n* = 6	IP injection of 0.1 ml/g 4.25% glucose PDF daily for 6 weeks.	200 µg/kg for 30 days	Reduced peritoneal thickness (*p* < 0.01 vs. PD); increased ultrafiltration (*p* < 0.01 vs. PD)	Inhibited MMT, anti-fibrosis, anti-inflammation and anti-angiogenesis by miR-27a-3p/TP53 pathway	[110]
ASCs	/	IP	Wistar Rat; male; 10–12 weeks; 280–320 g	*n* = 8	IP injection of 0.1% CG daily for 30 days	30 µg (4 × 10^11^ particles, on days 3 and 10); 30 days	Reduced peritoneal thickness (*p* < 0.01 vs. PF); Increased ultrafiltration (*p* < 0.01, vs. PF)	Anti-fibrosis by TGF-β/Smad pathway	[111]
PDE	/	IP	C57Bl/6J mice; 7–8 weeks	*n* = 8	IP injection of 0.3 ml 0.1% CG daily for a week	50 μg (on days 1 and 4); 7 days	Reduced peritoneal submesothelial thickness (*p* < 0.001, vs. PF)	Anti-fibrosis by PDGF-B/TGF-β pathway	[15]
Culture supernatant of P-Epi	/	IP	BALB/c nu/nu mice; male; 7–8 weeks	*n* = 6	Abdominal midline incision and scraping of the right and left parietal and visceral peritoneum evenly 33 times	1 ml; 14 days	Reduced peritoneal thickness	Anti-fibrosis	[112]
MCs	ILK	IP	C57BL/6 mice; male; 8 weeks	*n* = 5	IP injection of 0.1 ml/g 4.25% PDF daily for 6 weeks.	100 μg, 3 times a week for 5 weeks	Increased peritoneal submesothelial thickness (*p* < 0.001)	Pro-fibrosis by ILK/p38/TGF-β1 pathway	[113]
Macrophage	miR-204-5p	IP	Sprague-Dawley rats; male; 180–200 g	*n* = 5	IP injection of 100 ml/kg 4.25% glucose PDF daily for 4 weeks.	300 µg total protein (dissolved in 300 µl PBS), 3 times a week for 4 weeks	Reduced peritoneal thickness	Inhibited MMT by Foxc1/β-catenin pathway	[114]
DSS-MSCs	Hsa-miR-27a-5p_R-1	IP	C57BL/6 J mice	*n* = 6	IP injection of 3 ml 4.25% PDF daily for 4 weeks.	15 µg/days; 4 weeks	Reduced peritoneal thickness (*p* < 0.0001 vs. PF; *p* < 0.05 vs. Exo); increased ultrafiltration (*p* < 0.0001, vs. PF; *p* < 0.05 vs. Exo)	Anti-fibrosis by hsa-miR-27a-5p_R-1-STAT3-SHANK2 and STAT3/HIF-1α/VEGFA pathway	[115]

BMSC: bone marrow mesenchymal stem cell; IP: intraperitoneal injection; BMSC-exos: bone marrow mesenchymal stem cell-derived exosomes; PDF: peritoneal dialysis fluid; PBS: phosphate-buffered saline (control group); PD: peritoneal dialysis; PD + BMSC-exos: PD model with BMSC-exos treatment group; TP53: tumour protein 53; PD + BMSC-exos + TP53: PD model with BMSC-exos and TP53 cotreatment group; ASCs: adipose tissue stem cells; CG: chlorhexidine gluconate; PF: peritoneal fibrosis; ASCs-EVs: adipose tissue stem cells-derived extracellular vesicles; PDE: peritoneal dialysis effluent; PDE-EV: peritoneal dialysis effluent-derived extracellular vesicle; PDGF-B: platelet-derived growth factor subunit B; P-Epi: human epithelial-like peritoneal mesothelial cell; CS-P-Epi: supernatant collection from P-Epi; CS-P-Fibro: supernatant collection from P-Fibro; ILK: integrin-linked kinase; AS-IV-exos: lipopolysaccharides-stimulated and Astragaloside IV treated macrophage-derived exosomes; MMT: mesothelial-mesenchymal transition; DSS-MSCs: Danshenshin preconditioning mesenchymal stem cell; Exo: exosome.

**Table 3.** Cellular experiments on the role of extracellular vesicles in peritoneal fibrosis.

Origin of EVs	Cargoes	Methods	Species	Dosage; timing	Results	Mechanisms/pathways	References
hUC-MSCs	lncRNA CDHR	Co-culture	HPMCs	/; 48h	Decreased the expression of α-SMA and Vimentin and increased the expression of E-cadherin and PTEN and AKT/FOXO3a	Inhibited MMT by lncRNA CDHR / miR-3149/PTEN and AKT/FOXO	[113]
hUC-MSCs	lncRNA GAS5	Co-culture	HPMCs	/	Decreased expression of α-SMA and waveform protein and increased expression of E-cadherin	Inhibited MMT by lncRNA GAS5/miR-21/PTEN and Wnt/β-catenin	[114,115]
PDE	/	Co-incubation	P-MC, PDE-FB, P-FB	4.5 × 10^10^/ml and 0.45 × 10^10^/ml; 24h	Inhibit peritoneal cell proliferation (*p* < 0.0001 vs. PDE; *p* < 0.001 vs. PDGF-B)	Anti-fibrosis by PDGF-B/TGF-β pathway	[15]
Culture supernatant of P-Epi	/	Co-incubation	3T3 fibroblast cells	5 μL; 24h	Improved TGF-β-induced upregulation of type I collagen and fibronectin	Anti-fibrosis	[121]
MCs	ILK	Co-incubation	Fibroblast cells	30 μg/ml; 12 h	Increased fibronectin (*p* < 0.001), COL1A1 (*p* < 0.05), α-SMA (*p* < 0.05), and FAP protein (*p* < 0.05) expression in fibroblasts	Pro-fibrosis by ILK/p38/TGF-β1 pathway	[120]
MCs	miR-769-5p	Drug loading and co-incubation	HPMCs	10 μg	Reduced TGFBRI, PAI-1, SMAD2 and SMAD3 mRNA and protein expression	Anti-fibrosis by WT1-miR-769-5p pathway	[129]
Macrophage	miR-204-5p	Co-culture	PMC	1/2.5/5 μg; 48 h	Reduced expression of Vimentin, E-cadherin and α-SMA in RPMCs (*p* < 0.05, vs. LPS-exos)	Inhibited MMT by Foxc1/β-catenin pathway	[130]
DSS-MSCs	Hsa-miR-27a-5p_R-1	Co-incubation	HPMCs	1/3/5 μg/ml; 48 h	Decreased mRNA levels of fibrosis markers fibronectin and VEGFA and increased E-cadherin levels (*p* < 0.0001, vs. PF at 5 μg/ml)	Anti-fibrosis by hsa-miR-27a-5p_R-1-STAT3-SHANK2 and STAT3/HIF-1α/VEGFA pathway	[131]

hUC-MSCs: human umbilical cord mesenchymal stem cells; HPMCs: human peritoneal mesothelial cells; PTEN: phosphatase and tensin homolog deleted on chromosome ten; MMT: mesothelial-mesenchymal transition; PDE: peritoneal dialysis effluent; P-MC: human primary peritoneal mesothelial cells; P-FB: human primary fibroblast from peritoneal biopsies; PDE-FBs: human primary fibroblast from PDE; PDGF: platelet-derived growth factor; P-Epi: human epithelial-like peritoneal mesothelial cell; MCs: mesothelial cells; ILK: integrin-linked kinase; RPMCs: rat peritoneal mesothelial cells; LPS: lipopolysaccharides; DSS-MSCs: Danshenshin preconditioning mesenchymal stem cell; VEGFA: vascular endothelial growth factor A.

### 5.1. Mesenchymal stem cell-derived EVs (MSC-EVs)

MSCs, which are multipotent adult stem cells isolated from diverse biological sources, such as bone marrow, adipose tissue, and the umbilical cord, exhibit anti-inflammatory, immunomodulatory, and antifibrotic properties [110]. Emerging evidence indicates that MSC-derived EVs may ameliorate PF through multiple mechanisms.

Bone marrow mesenchymal stem cell-derived exosomes (BMSC-Exos) have been shown to attenuate PF by inhibiting MMT, inflammation, and angiogenesis. Mechanistically, these effects are mediated by exosomal delivery of miR-27a-3p, which directly targets and downregulates TP53 expression, leading to preservation of the peritoneal membrane ultrastructure, maintenance of ultrafiltration capacity, and protection of peritoneal functional integrity [111,112].

A research team led by Lina Yang identified the human umbilical cord mesenchymal stem cell exosomes (hUC-MSC-Exos) exert antifibrotic effects through two distinct lncRNAs [113–115]. The lncRNA CDHR regulates AKT/FOXO signalling *via* the miR-3149/PTEN axis, while the lncRNA GAS5 blocks Wnt/β-catenin signalling *via* the miR-21/PTEN axis. Both pathways converge to suppress MMT by modulating key phenotypic markers (such as E-cadherin, vimentin and α-SMA) [113–115]. These complementary mechanisms suggest potential synergistic therapeutic strategies that combine the lncRNA CDHR and GAS5 to simultaneously target PTEN-mediated pathways and enhance MMT reversal.

Adipose tissue stem cell-derived EVs (ASC-EVs) modulate the TGF-β/Smad axis by downregulating profibrotic effectors (TGF-β, Smad3) and upregulating Smad7, a critical endogenous inhibitor of TGF-β signalling. These molecular changes correlated with measurable histological improvements, including diminished infiltration of proinflammatory M1 macrophages and leukocytes, as well as inhibition of maladaptive angiogenesis, evidenced by reduced capillary density and VEGF expression levels. Collectively, these coordinated effects resulted in reduced peritoneal thickness and enhanced ultrafiltration functionality compared with those of the PF group [116].

Notably, MSC-EVs possess low immunogenicity and tumorigenicity, which ensures their superior biosafety in therapeutic applications, and they also exhibit targeting ability towards injured peritoneal tissues, facilitating the precise delivery of their bioactive cargoes [114,117].

### 5.2. Peritoneal dialysis effluent-derived EVs

Peritoneal dialysis effluent, a readily accessible biofluid obtained noninvasively from PD patients, has emerged as a valuable source for molecular profiling [103,106,118,119]. Multiple approaches, including fluorescent labelling, nanoparticle tracking analysis, and proteomic profiling, have verified that this biofluid is rich in EVs [103,106,118,119].

A mechanistic investigation demonstrated that peritoneal dialysis effluent-derived exosomes (PDE-Exos) from paediatric patients exhibit potent antifibrotic effects *via* dual-pathway inhibition of platelet-derived growth factor B (PDGF-B) and TGF-β signalling. *In vitro*, PDE-Exos significantly inhibited peritoneal cell proliferation, MMT, collagen synthesis, and the TGF-β-induced upregulation of type I collagen and fibronectin. *In vivo*, they preserved the mesothelial layer structure and reduced submesothelial matrix deposition. This dual-axis inhibition mechanism positions PDE-Exos as promising endogenous nanotherapeutics for peritoneal membrane preservation [15].

### 5.3. Mesothelial cell-derived EVs

Mesothelial cells, the primary functional cells of the peritoneum, undergo damage and secrete profibrotic factors under long-term PD [120]. Interestingly, mesothelial cell-derived EVs exhibit context-dependent functional phenotypes, with both antifibrotic and profibrotic effects reported.

A study using culture supernatant from human epithelial-like peritoneal mesothelial cells (P-Epi) reported that P-Epi-derived exosomes exert antifibrotic effects on TGF-β-stimulated 3T3 fibroblasts. They suppressed ECM accumulation (collagen I synthesis and fibronectin deposition), and pathological structural changes (peritoneal adhesions, peritoneal thickness, and the fibrotic markers α-SMA and TIMP-1) [121].

Conversely, under mechanical injury or inflammatory stimulation, mesothelial cells secrete integrin-linked kinase (ILK)-rich EVs. These EVs are internalised by fibroblasts, activating the p38 MAPK pathway and increasing TGF-β1 synthesis. The newly generated TGF-β1 forms a positive feedback loop, amplifying both the release of ILK-rich EVs from mesothelial cells and fibroblast activation. Compared with control EVs, ILK-rich EVs upregulated multiple profibrotic markers (fibronectin, COL1A1, α-SMA, and FAP) *in vitro* and increased submesothelial peritoneal thickness *in vivo*. Intervention experiments confirmed that blocking EV secretion *via* GW4869 (a non-competitive phospholipase inhibitor) and Rab27a (a small GTPase) knockdown significantly inhibited PF [120].

As native EVs derived from peritoneal mesothelial cells, MC-EVs have inherent targeting to peritoneal cells, but their context-dependent profibrotic properties under inflammatory or mechanical injury conditions pose potential risks for therapeutic applications.

Overall, the dual role of EVs in PF—mediating both profibrotic and antifibrotic processes—has spurred the development of therapeutic strategies that either utilise or target EVs. Notably, natural EVs-based therapies for PF present their origin-specific advantages and limitations: MSC-EVs offer multitarget therapeutic actions and superior biosafety (low tumorigenicity and immunogenicity) [114,117], although challenges in large-scale production, standardisation, and source-dependent heterogeneity remain [122]; PDE-EVs contain endogenous antifibrotic components and hold promise as diagnostic biomarkers, yet robust therapeutic validation is lacking [51,123–126]; MC-EVs, naturally present in the peritoneum, can directly target peritoneal cells but carry a context-dependent profibrotic risk [120,127]. Head-to-head comparison of these EV types in PF models is urgently needed to clarify their relative therapeutic efficacy and safety profiles. These limitations have motivated the exploration of functionalised EVs to overcome these challenges.

## 6. Role of functionalized EVs for PF

### 6.1. Cargo-loading derived EVs

Given their great potential as drug delivery vehicles, multiple approaches have been developed to load bioactive molecules into EVs in preclinical and clinical studies [128]. In a landmark study, mesothelial cell-derived EVs loaded with miR-769-5p represent a novel antifibrotic strategy. This EV‑mediated delivery upregulated miR-769-5p in mesothelial cells *via* modulating the HDAC1/WT1 axis. This intervention suppressed the expression of fibrotic markers, including Smad2, Smad3, TGFBRI, and PAI-1, ultimately reversing MMT and mitigating subepithelial thickening. Notably, EV encapsulation enhances miR-769-5p stability and tissue-specific accumulation, addressing critical challenges of conventional RNA-based therapies for PF [129].

### 6.2. Drug preconditioning-derived exosomes

Preconditioning cells with pharmacological agents can enhance the therapeutic properties of their derived EVs. This strategy induces cellular resistance to stress, thereby endowing the secreted EVs with additional therapeutic functions or improved therapeutic efficacy [93]. Recent investigations have successfully modulated EV functions through specific preconditioning strategies.

For instance, Shan et al. identified a dual therapeutic action of astragaloside IV (AS-IV), a saponin derived from the traditional Chinese herb *Astragalus membranaceus*, in PF management. In addition to its direct anti-inflammatory properties, AS-IV mediates intercellular communication by reprogramming macrophage-derived exosomes. Specifically, it targets the Foxc1/β-catenin signalling axis *via* macrophage-derived exosome-mediated delivery of miR-204-5p, effectively mitigating PF and suppressing the abnormal expression of pathological hallmarks, including Vimentin, E-cadherin, α-SMA and collagen I in rat peritoneal mesothelial cells (*p* < 0.05, vs. the LPS-stimulated macrophage-derived exosome group) [130].

Similarly, the therapeutic potential of exosomes derived from pre-treated MSCs has been explored. Danshensu (DSS), a bioactive phytochemical extracted from the herb *Salvia miltiorrhiza* (Danshen), is used to precondition MSCs due to its significant antifibrotic properties. A recent study by Liang et al. revealed that exosomes derived from DSS-pre-treated MSCs (DSS-Exos) ameliorate PF through a defined molecular mechanism. Mechanistically, DSS-Exos regulate angiogenesis and fibrotic tissue remodelling *via* the hsa-miR-27a-5p_R-1-STAT3-SHANK2 axis and the STAT3/HIF-1α/VEGFA signalling axis. Compared with the PF model group and the untreated MSC‑Exo group, DSS-Exos significantly reduced peritoneal thickness and improved ultrafiltration capacity. These findings highlight the synergistic potential of integrating modern exosome technology with traditional Chinese medicine [131].

In conclusion, functionalised EVs offer a promising approach to address the limitations of natural EVs. By incorporating exogenous cargo or modifying parent cells, these engineered EVs enhance peritoneal targeting specificity, improve cargo stability, and optimise delivery efficiency. However, research on functionalised EVs in PF remains scarce. In particular, studies on EV-based gene editing and targeted molecular modification for PF therapy are still insufficient. Meanwhile, their clinical translation is hindered by technical challenges and insufficient safety validation [132].

## 7. Current challenges and potentials

With extensive investigations into EVs, their potential therapeutic value in PF has been progressively elucidated. However, the development of EV-based therapeutics for PF faces three interrelated challenges requiring systematic resolution.

### 7.1. Technical barriers

The clinical translation of EV-based therapies for PF is hindered by technical hurdles in production. Current isolation methods, particularly ultracentrifugation, suffer from low yield and insufficient purity due to co-isolated contaminants such as non-vesicular extracellular particles and protein aggregates [133,134]. Beyond yield and purity, inter-batch reproducibility remains a critical challenge, as subtle variations in cell culture conditions, isolation procedures, or storage protocols can lead to significant heterogeneity in EV size, cargo composition, and functional potency [133,135].

The MISEV 2023 guidelines provide a systematic framework to address these issues [133]. They recommend using complementary separation techniques to balance yield and purity, employing orthogonal methods for rigorous characterisation, and transparently reporting all procedural parameters to enhance experimental reproducibility and facilitate cross-study comparability. However, despite these guidelines, the scalability of current isolation technologies under Good Manufacturing Practice (GMP) conditions remains to be fully validated. Standardised protocols for EV characterisation and potency testing, addressing critical quality attributes from donor variation to final product formulation, are urgently needed to facilitate regulatory harmonisation and clinical translation [136].

### 7.2. Biodistribution and stability barriers

The unfavourable *in vivo* fate of systemically administered EVs poses a major barrier to their therapeutic application in peritoneal fibrosis. They are susceptible to rapid degradation and clearance by the mononuclear phagocyte system, exhibiting poor circulatory stability with a short half-life [137,138]. Moreover, most EVs accumulate in the liver, spleen, and lungs, with only a minimal fraction reaching the peritoneal tissue [137]. Furthermore, unmodified EVs lack intrinsic targeting specificity, leading to off-target effects and compromised therapeutic efficacy [122,138].

Emerging evidences offer promising solutions to these pharmacokinetic limitations. Surface modification with immunomodulatory proteins (e.g. CD47) enables immune evasion *via* the “don’t eat me” mechanism [139]; polymer conjugation (e.g. POxylation) enhances circulatory stability and tissue accumulation [140]; and hydrogel encapsulation facilitates sustained local delivery [141]. Despite promising advances in other disease models, the fate of EVs within the peritoneal niche remains poorly understood in PF, representing a critical knowledge gap.

### 7.3. Immunogenicity and oncogenicity barriers

Despite their lower immunogenicity compared with parental cells, EVs still pose toxicity and adverse effects. For instance, systemically delivered EVs and their bioactive components may induce immunomodulation and unintended cytotoxicity [122]. Allogeneic EVs, in particular, highly express donor MHC molecules that can trigger host immune responses, leading to graft rejection or toxicity [136]. Compounding these immunological risks is the potential oncogenicity: EVs carrying pro-tumorigenic cargo or originating from malignant cells may inadvertently promote unwanted cellular proliferation [142–144].

Mitigation strategies encompass multiple levels of intervention. At the cellular source level, the use of low-immunogenicity cells (e.g. MSCs) ensures that EVs inherit favourable immunological properties from their parent cells [145,146]. Genetic engineering can further reduce the risk of T-cell-mediated rejection by equipping EVs with enhanced immunoregulatory functions [146,147]. At the administration level, optimised delivery routes (e.g. local injection or hydrogel encapsulation) can mitigate systemic exposure and the need for repeated dosing [148]. Beyond these, comprehensive preclinical toxicology studies in relevant large animal models are essential prerequisites for clinical translation.

In summary, the clinical translation of EV-based therapies for PF requires coordinated efforts to overcome three interconnected barriers: scalable and reproducible production, favourable pharmacokinetics and biodistribution, and proven long-term safety. While engineering strategies offer promising solutions to each challenge, their integration into a unified therapeutic platform remains a key research priority. Systematic validation in preclinical models, coupled with adherence to evolving regulatory guidelines, will pave the way for EV-based therapeutics to realise their clinical potential in PF.

## 8. Future perspectives

Peritoneal fibrosis remains a persistent challenge in patients undergoing peritoneal dialysis, impairing treatment efficacy and imposing substantial clinical and economic burdens. In this review, we highlight that EVs derived from various cellular sources exert predominantly therapeutic effects in peritoneal fibrosis, while limited evidence supports their potential involvement as pathogenic mediators. Through precise delivery of diverse cargoes (e.g. proteins, miRNAs, lncRNAs, DNA, lipids, and metabolites), EVs from various origins coordinately regulate intercellular communication. They exhibit pleiotropic effects against key pathological processes in PF, including inflammation, fibroblast activation, mesothelial-to-mesenchymal transition, ECM imbalance, and aberrant angiogenesis. Of note, EVs derived from distinct cellular sources often target overlapping signalling pathways and act synergistically to augment their antifibrotic effects. Conversely, certain EVs can also promote fibrogenesis, conferring a “double-edged sword” role in the progression of PF. This dual function underscores the critical regulatory role of EVs in PD-associated PF and the need for precise modulation of EV‑mediated networks. Furthermore, profiling EV cargo in biofluids holds great promise for the early diagnosis and dynamic monitoring of PF.

Despite their potential, EV-based therapeutic strategies for PF face multifaceted challenges, including technical barriers in preparation, suboptimal *in vivo* biodistribution, and long-term safety concerns. Most current studies are limited to preclinical animal models with short-term observation, lacking comprehensive safety assessments in clinical trials. In addition, key pharmacological parameters, including peritoneal retention dynamics, metabolic fate, dose–response relationships, and immunogenicity under chronic exposure, are poorly characterised. Advancing clinical applicability requires rigorous clinical trials to establish safety–efficacy profiles and mechanistic studies to elucidate synergies between EVs and conventional therapies. Integrated multiomics approaches are essential for dissecting multitarget crosstalk and optimising precision medicine strategies for PF.

On the diagnostic front, clinical translation is already underway. Notably, multiple registered clinical trials are investigating the diagnostic and monitoring potential of extracellular vesicles in kidney disease: as an assessment tool for allograft fibrosis after kidney transplantation (NCT03870542), a dynamic monitoring indicator for haemodialysis (NCT05957146), and a biomarker for chronic renal failure (NCT04700631). These studies underscore the versatile potential of EVs in nephrology and provide valuable clinical experience that may ultimately inform the development of EV-based therapeutics.

Collectively, EVs represent a transformative “cell-free therapy” for peritoneal fibrosis, bridging the gap between preclinical research and clinical application. Their multitargeted regulatory properties enable the reversal of fibrotic progression and the restoration of peritoneal function, offering new hope for PD patients.

## Data Availability

No datasets were generated or analysed during the current study.
